# Deep Learning-Based Computer-Aided Detection and Diagnosis System for Malignant Biliary Stricture (With Video) †

**DOI:** 10.3390/cancers18152410

**Published:** 2026-07-26

**Authors:** Qingyu Tang, Sanping Zhou, Zizhan Tang, Kangpeng Li, Zejian Huang, Lei Zhang, Dapeng Bian, Qiushi Feng, Qi Li, Hao Sun, Jie Tao, Le Wang, Zhimin Geng, Chen Chen

**Affiliations:** 1 Department of Hepatobiliary Surgery, The First Affiliated Hospital of Xi’an Jiaotong University, Xi’an 710061, China; tangqingyu@stu.xjtu.edu.cn (Q.T.); 2203211142@stu.xjtu.edu.cn (K.L.); docliqi@xjtufh.edu.cn (Q.L.); sunhaoxjyf@126.com (H.S.); jxph062747@sohu.com (J.T.); 2 National Key Laboratory of Human-Machine Hybrid Augmented Intelligence, National Engineering Research Center for Visual Information and Applications, Institute of Artificial Intelligence and Robotics, Xi’an Jiaotong University, Xi’an 710049, China; spzhou@xjtu.edu.cn (S.Z.); lewang@xjtu.edu.cn (L.W.); 3 USC Viterbi School of Engineering, University of Southern California, Los Angeles, CA 90089, USA; mtang410@usc.edu; 4 Department of Hepatobiliary Surgery, Sun Yat-Sen Memorial Hospital, Sun Yat-Sen University, Guangzhou 510120, China; hzej@mail.sysu.edu.cn (Z.H.); zhangl9@mail.sysu.edu.cn (L.Z.); 5 Department of Hepatobiliary Surgery, Peking University First Hospital, Beijing 100034, China; 13810584377@126.com (D.B.); fqschina@sina.com (Q.F.)

**Keywords:** artificial intelligence, bile duct neoplasms, cholangioscopy, deep learning, computer-assisted diagnosis

## Abstract

Background/Objectives: Malignant biliary stricture (MBS) remains difficult to diagnose accurately despite digital single-operator cholangioscopy (DSOC). We developed a deep learning (DL)-based computer-aided detection (CADe) and diagnosis (CADx) system for DSOC-based MBS assessment. Methods: This retrospective multicenter study included 149 patients from one center for model development and internal validation and 25 patients from two independent centers for external evaluation. CADe used a You Only Look Once version 11 (YOLOv11) architecture to localize irregular mucosa, abnormal vasculature, and nodular protrusions defined by the Carlos Robles-Medranda and Mendoza criteria. CADx used a Residual Network-18 classifier with gradient-weighted class activation mapping for interpretability. Results: CADe achieved a mean average precision at 50% intersection-over-union of 91.2%, with a precision of 92.0% and recall of 87.0%. CADx achieved a frame-level area under the receiver operating characteristic curve (AUC) of 0.960 in internal validation and 0.843 in external validation. External frame-level sensitivity was 52.0% and specificity was 95.2%. For the patient-level external endpoint, sensitivity was 85.7%, specificity was 94.4%, accuracy was 92.0%, and AUC was 0.881. Conclusions: The two-stage system combines localization of predefined cholangioscopic features with interpretable diagnostic classification. The small external cohort and marked reduction in frame-level sensitivity preclude firm conclusions regarding generalizability; prospective multicenter and live-procedure evaluation is required.

## Simple Summary

Malignant biliary stricture is difficult to detect early, and current tests often miss it or give uncertain answers. Doctors can pass a thin camera directly into the bile duct to inspect narrowed areas, but reading these images is challenging and varies from one specialist to another. We developed a two-stage artificial intelligence system that highlights predefined suspicious features, classifies images, and shows which regions influenced its prediction. The system was evaluated retrospectively using images and archived videos from three hospitals. External sensitivity was lower for individual frames, while patient-level estimates from only 25 external patients were imprecise. Prospective testing on uncurated live procedures is therefore required before clinical use.

## 1. Introduction

The early detection and accurate diagnosis of malignant biliary stricture (MBS) are critical for optimizing clinical outcomes, as delayed diagnosis contributes to poor prognosis and limited therapeutic options [1,2]. Digital single-operator cholangioscopy (DSOC) has emerged as a minimally invasive modality offering direct, high-resolution visualization and therapeutic intervention within the pancreatobiliary system. However, its diagnostic accuracy remains suboptimal compared to its technical potential [3]. Conventional methods, including endoscopic retrograde cholangiopancreatography (ERCP)-guided brush cytology and forceps biopsy, serve as first-line techniques for evaluating indeterminate strictures without visible masses [4]. Yet their sensitivity is limited (23–81%), with diagnostic performance heavily dependent on endoscopist expertise [3,5].

DSOC theoretically overcomes these limitations by enabling targeted tissue sampling under direct visualization [6]. Nevertheless, persistent challenges include interobserver variability in image interpretation and a clinically significant false-negative rate. DSOC-guided biopsies exhibit modest sensitivity (~74%), undermining diagnostic reliability [7,8]. The morphological complexity and heterogeneity of biliary lesions, compounded by the absence of standardized diagnostic criteria, further exacerbate these issues [9].

Deep learning (DL), particularly in convolutional neural networks (CNNs), has demonstrated substantial potential in endoscopic image analysis by autonomously extracting discriminative features and reducing reliance on subjective interpretation [10,11]. Prior DSOC AI studies have progressed from static-image and video-based models to multicenter validation, morphologic-feature characterization, and prospective real-time use [12,13,14,15,16,17,18,19,20]. However, explicit bounding-box localization of named cholangioscopic features before a separate diagnostic classification stage, together with adequately powered external patient-level evaluation, remains insufficiently studied [21,22]. The ‘black box’ nature of DL models also raises concerns about diagnostic accountability, highlighting the need for interpretable AI-assisted decision support [23].

To address these gaps, we developed a two-stage DL framework using 5266 quality-controlled images from 149 DSOC patients. Stage I used YOLOv11 to localize three malignancy-associated cholangioscopic features based on the Carlos Robles-Medranda (CRM) and Mendoza criteria [4,24]. Stage II used ResNet18 [25] with Grad-CAM [26] and Grad-CAM++ [27] to classify images as neoplastic or non-neoplastic and visualize regions contributing to the prediction. The locked system was subsequently evaluated in 25 patients from two external centers. The source code is available at https://github.com/ZizhanT/dsoc-lesion-detection (accessed on 23 July 2026).

## 2. Materials and Methods

### 2.1. Study Design

This retrospective multicenter study was reported in accordance with the STROBE statement and was not prospectively registered [28]. Model development and internal validation were performed using 5266 quality-controlled static images from 149 DSOC examinations at the First Affiliated Hospital of Xi’an Jiaotong University (FAH-XJTU). Stage I developed a CADe model for localization of three predefined malignancy-associated morphological features. Stage II developed a CADx model for binary classification of neoplastic versus non-neoplastic images. The locked system was subsequently evaluated using 45 archived videos from 25 patients at two external centers.

The CADe feature annotations and the CADx reference diagnosis served different purposes. CADe labels represented the presence of malignant-appearing morphological features in individual images. Such features could be present in patients whose final reference diagnosis was non-neoplastic. CADx ground truth was assigned at the patient level from histopathology or a composite clinical reference standard based on at least 6 months of follow-up. Thus, malignant-appearing image features did not by themselves determine the final neoplastic/non-neoplastic group.

An online demonstration was developed to visualize the outputs of the pretrained CADe and CADx models on uploaded DSOC video clips. The platform is presented as a research demonstration rather than a validated clinical service.

### 2.2. Study Population

A total of 174 patients from three centers were included. For model development and internal validation, 159 consecutive patients underwent DSOC at FAH-XJTU between June 2021 and June 2025. After application of the exclusion criteria, 149 patients were eligible. The final reference diagnosis classified 74 patients as neoplastic and 75 as non-neoplastic. Surgical histopathological confirmation was available in 107 patients (71.8%), including 66 of 74 neoplastic patients and 41 of 75 non-neoplastic patients. No patient was classified solely on the basis of endoscopic biopsy or cytology. The remaining 42 patients (28.2%), including 8 neoplastic and 34 non-neoplastic patients, were classified using a composite clinical reference standard incorporating at least 6 months of follow-up, serial imaging, tumor markers, and clinical course.

For external evaluation, all model parameters were locked before analysis. The cohort comprised 25 patients: 12 from Sun Yat-sen Memorial Hospital, Sun Yat-sen University, Guangzhou, and 13 from Peking University First Hospital, Beijing. Seven patients had a neoplastic reference diagnosis and 18 had a non-neoplastic reference diagnosis. All 25 external patients had surgical histopathological confirmation.

All patients were ≥18 years old, referred for DSOC evaluation of suspected biliary neoplasia or strictures, and had documented DSOC procedural records. Exclusion criteria included: (a) inadequate image or video quality precluding reliable visual assessment; (b) absence of definitive histopathological diagnosis or follow-up-based clinical confirmation; (c) absence of biliary strictures despite undergoing DSOC for suspected biliary stricture examination; and (d) intraductal papillary mucinous neoplasm of bile duct (IPMN-B).

### 2.3. DSOC Procedure

DSOC procedures were performed by expert biliary endoscopists (annual DSOC volume >100 cases). Patients received general anesthesia with prophylactic antibiotics and were positioned laterally. Selective biliary cannulation was achieved using an Olympus TJF-260V duodenoscope, followed by endoscopic sphincterotomy when indicated. The DSOC system (Micro-Tech Medical) was introduced through the working channel. During systematic biliary tree evaluation, bile and contrast medium were aspirated, and continuous sterile saline irrigation was applied to optimize visualization. The catheter was advanced to tertiary biliary branches and gradually withdrawn under direct visualization to inspect mucosal patterns. Sectoral duct exploration utilized guidewire-assisted techniques. Targeted biopsies were obtained using disposable forceps when suspicious lesions were identified. The entire procedures were recorded using the Bandicam system.

### 2.4. Development and Evaluation Procedures for the DL-Based MBS CADe and CADx System

Stage I (CADe—lesion detection): Archived DSOC videos from the 149 eligible FAH-XJTU patients underwent standardized manual frame selection and quality control. Two biliary endoscopists reviewed and annotated irregular vascular patterns, abnormal mucosal surfaces, and nodular protrusions according to the CRM and Mendoza criteria using Roboflow. The annotations described image-level morphology and were independent of the final patient-level reference diagnosis. Candidate YOLOv5, YOLOv8, YOLOv11, and YOLOv12 detectors were evaluated using the same patient-level partition. YOLOv11 provided the highest overall mAP50 and mAP50–95 (Table S5). The 149-patient development cohort was split at the patient level into a training set of 134 patients (4739 images) and an internal validation set of 15 patients (527 images). All images from a patient were restricted to one partition. The validation set was used for early stopping, checkpoint and model selection, and operating-threshold calibration. It was therefore not an independent internal test set. No external patient, image, or video was used for model selection or threshold tuning.

Stage II (CADx—diagnosis classification): ResNet18 was used for binary image classification with the same 134-patient training and 15-patient validation partition. This compact backbone was selected to limit model complexity in a modest-sized clinical dataset, support efficient inference, and permit Grad-CAM/Grad-CAM++ visualization. No k-fold cross-validation was performed. The external cohorts were retained for independent evaluation after model and threshold locking.

Before consensus annotation, two biliary endoscopists independently reviewed all 5266 development images and assigned binary image-level labels as neoplastic or non-neoplastic while blinded to each other’s annotations. They agreed on 5217 images (99.07%), yielding an unweighted Cohen’s κ of 0.980. Reader 1 classified 32 of the 49 discordant images as neoplastic when Reader 2 classified them as non-neoplastic. The reverse occurred for 17 images. All discordant images were jointly reviewed and resolved by consensus. This image-review step did not replace the patient-level reference diagnosis as the clinical reference standard.

### 2.5. Statistical Methods

The CADe model was assessed at the image level using mean average precision at 50% intersection-over-union (mAP50), precision, recall, and F1-score. CADx discrimination was assessed using AUC, sensitivity, specificity, accuracy, positive predictive value (PPV), and negative predictive value (NPV). All metrics were identified as frame- or patient-level. Patient-level diagnostic performance was the external endpoint. External frame-level results were primary descriptive analyses because frames from the same patient are correlated. Patient-level scores were obtained as the mean of each patient’s top-scoring decile of frame probabilities, and the fixed threshold of 0.58 selected on the internal validation set was applied without external tuning. External frame-level confidence intervals and the patient-level AUC interval were computed using stratified patient bootstrap resampling (1000 resamples). Wilson intervals were used for patient-level diagnostic proportions and for internal frame-level diagnostic proportions.

All internal data partitions were created at the patient level. The external centers were not used for model selection or threshold tuning. This was a retrospective convenience sample of consecutive eligible DSOC cases, and no a priori sample-size calculation was performed. Baseline categorical variables were compared using the chi-square test or Fisher’s exact test, as appropriate. Continuous variables were summarized as median and interquartile range values and compared using the Mann–Whitney U test. All tests were two-sided with alpha = 0.05.

Low-quality frames were excluded manually before model analysis according to predefined visual criteria, including severe blur, inadequate illumination, extensive bile or debris, and substantial instrument occlusion. This manual quality-control step was not treated as missing-data imputation. No automated quality-control module was developed, validated, or applied in the present study. A candidate future preprocessing module could combine variance of the Laplacian for sharpness, mean image intensity for exposure, and the fraction of near-saturated pixels. Its thresholds would require center-specific calibration and prospective validation. The current results therefore do not represent a fully automated real-world video pipeline.

### 2.6. Interpretability Analyses and Exploratory Reader Study

To enhance interpretability, advanced Grad-CAM++ and Grad-CAM algorithms were applied to visualize the critical attention regions identified by the DL-based CAD system. Heatmap analysis quantified the concordance between the CNN-based classification model’s attention regions and the biliary neoplasia-related features (irregular vascular patterns, aberrant mucosal surfaces, and nodular protrusions) prioritized by the CRM and Mendoza diagnostic standards. Misclassification analysis was conducted to identify potential areas for model improvement. Feature map visualization illustrates the distinct features extracted by different layers of ResNet18 contributing to the final diagnosis decision.

An exploratory reader experiment presented the same set of sampled static images to the CADx model and two expert biliary endoscopists (annual DSOC volume > 100 procedures), who were blinded to clinical and pathological data and model predictions. The readers were additionally allowed to review the corresponding 5 s clips. Because the information inputs were not identical, the comparison was treated as descriptive. No formal superiority inference was made.

### 2.7. Development and Intended Use of the Online Demonstration Platform

An online demonstration system was developed to display CADe bounding boxes and CADx predictions for DSOC clips. No third-party uploaded data were used for model development or evaluation in this study. The present Institutional Review Board approval covers only the retrospective institutional datasets and does not authorize retention or secondary training use of external uploads. Accordingly, the platform is not presented as a research data-collection channel or a validated clinical service, and identifiable clinical data should not be submitted. Any future retention or reuse would require documented de-identification, access and retention controls, consent or an approved waiver, and separate ethics approval.

## 3. Results

### 3.1. Baseline Information of Participants

A total of 149 patients from FAH-XJTU were included, comprising 74 patients with a neoplastic reference diagnosis and 75 with a non-neoplastic reference diagnosis (Table 1). The median age was 63.0 years (IQR, 54.0–69.0), and 60 patients (40.3%) were female. Jaundice was more frequent in the neoplastic group (70.3% vs. 38.7%; *p* < 0.001). Total bilirubin, CEA, CA19-9, and CA125 were higher in the neoplastic group, whereas ALP, GGT, ALT, AST, and AFP did not differ significantly. Surgical pathology confirmed the reference diagnosis in 107 patients (71.8%). No patient was classified by endoscopic biopsy or cytology alone, and 42 (28.2%) were classified using the composite clinical reference standard. The non-neoplastic cohort included four documented cases of IgG4-related sclerosing cholangitis and two cases of autoimmune cholangitis without explicit IgG4 attribution. No primary sclerosing cholangitis was documented among the 146 patients with available Primary Sclerosing Cholangitis (PSC)-history data.

### 3.2. Development and Validation of the DL-Based MBS CADe and CADx System

**Figure 1:**
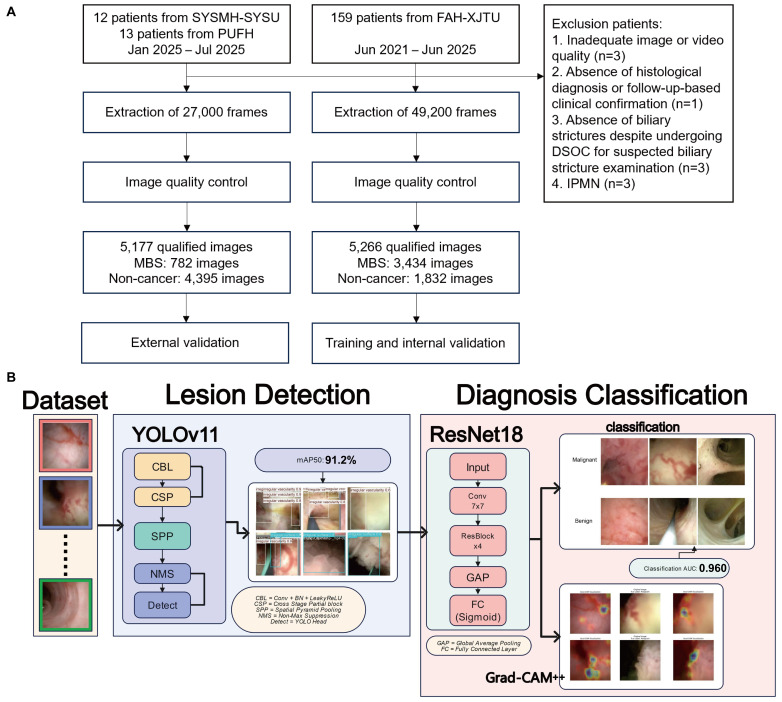
Schematic workflow of the deep learning-based computer-aided detection and computer-aided diagnosis system for malignant biliary stricture. (**A**) Workflow of patient cohorts and model development procedures. (**B**) Framework of the computer-aided detection and computer-aided diagnosis system.

A novel DL framework integrating CADe and CADx of MBS was developed utilizing our single-center dataset retrospectively (Figure 1B). The model exhibited stable convergence with monotonically decreasing training and validation losses across both phases, achieving a total loss of 2.27 (Figure S1A–C). The inference speed was approximately 2.7 ms per image.

#### 3.2.1. Stage I: CADe Performance

**Figure 2:**
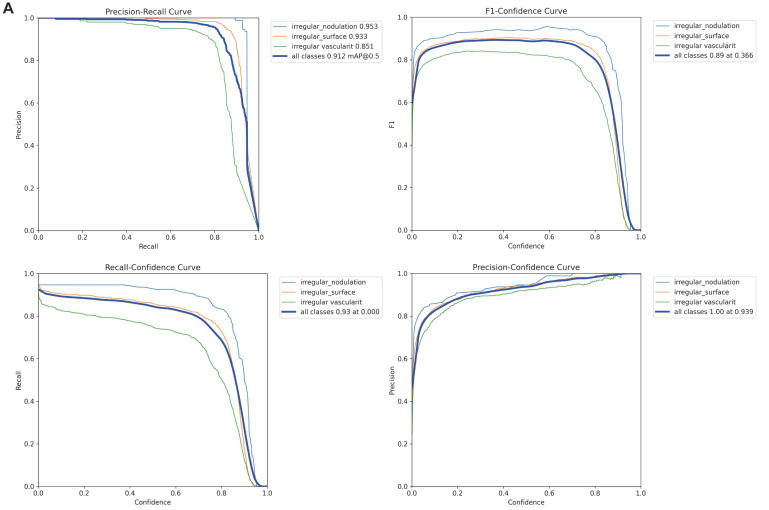

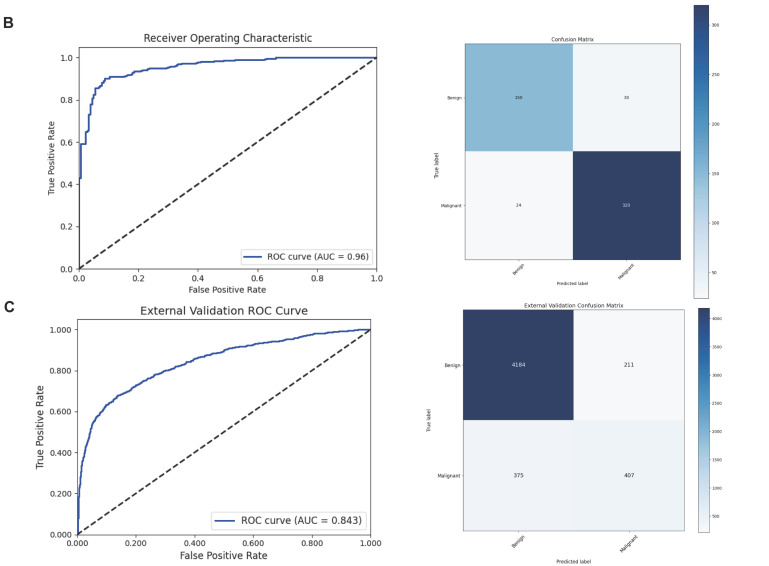
Detection and diagnosis performance of the deep learning-based computer-aided detection and computer-aided diagnosis system for malignant biliary stricture. (**A**) Precision–recall curve, F1–confidence curve, recall–confidence curve, and precision–confidence curve of the detection performance. (**B**,**C**) Receiver operating characteristic curves and confusion matrices of diagnosis performance in the internal and external validation cohorts, respectively. In (**C**), the external confusion matrix comprises 4184 true negatives, 211 false positives, 375 false negatives, and 407 true positives, together totaling the 5177 analyzed external frames (4395 benign and 782 malignant).

The CADe model attained a mAP50 of 91.2% (95% CI 85.4–97.0%) on internal validation (Table S1). Subgroup analysis demonstrated superior performance for nodular protrusion detection (mAP50: 95.3%) compared to surface irregularity (93.3%) and vascular irregularity (85.1%). Validation precision and recall were 92.0% and 87.0%, respectively, yielding an F1-score of 89.4% (Figure 2A).

#### 3.2.2. Stage II: CADx Performance

The CADx model achieved an AUC of 0.960 (95% CI 0.945–0.975) in internal validation (Figure 2B). The confusion matrix (TN = 150, FP = 33, FN = 24, TP = 320) yielded a sensitivity of 93.0%, specificity of 82.0%, accuracy of 89.2%, PPV of 90.7%, and NPV of 86.2% (Table 2).

#### 3.2.3. External Validation

External evaluation included 25 patients (7 neoplastic and 18 non-neoplastic) from two tertiary centers and comprised 45 archived DSOC videos. After manual quality control, 5177 frames were analyzed, including 782 malignant and 4395 benign frames. At the frame level, CADx achieved an AUC of 0.843, sensitivity of 52.0%, specificity of 95.2%, accuracy of 88.7%, PPV of 65.9%, and NPV of 91.8% (Table 2). The sensitivity–specificity imbalance may reflect threshold calibration, intercenter domain shift, case mix, manual frame selection, and possible overfitting. These contributions could not be quantified because standardized center-specific acquisition metadata were unavailable. For the patient-level endpoint, six of seven neoplastic and 17 of 18 non-neoplastic patients were correctly classified, yielding a sensitivity of 85.7% (95% CI 48.7–97.4), specificity of 94.4% (74.2–99.0), accuracy of 92.0% (75.0–97.8), PPV of 85.7%, NPV of 94.4%, and AUC of 0.881 (0.619–1.000) (Table S4, Figure S2).

### 3.3. Interpretability of the DL-Based MBS CADe and CADx System

**Figure 3:**
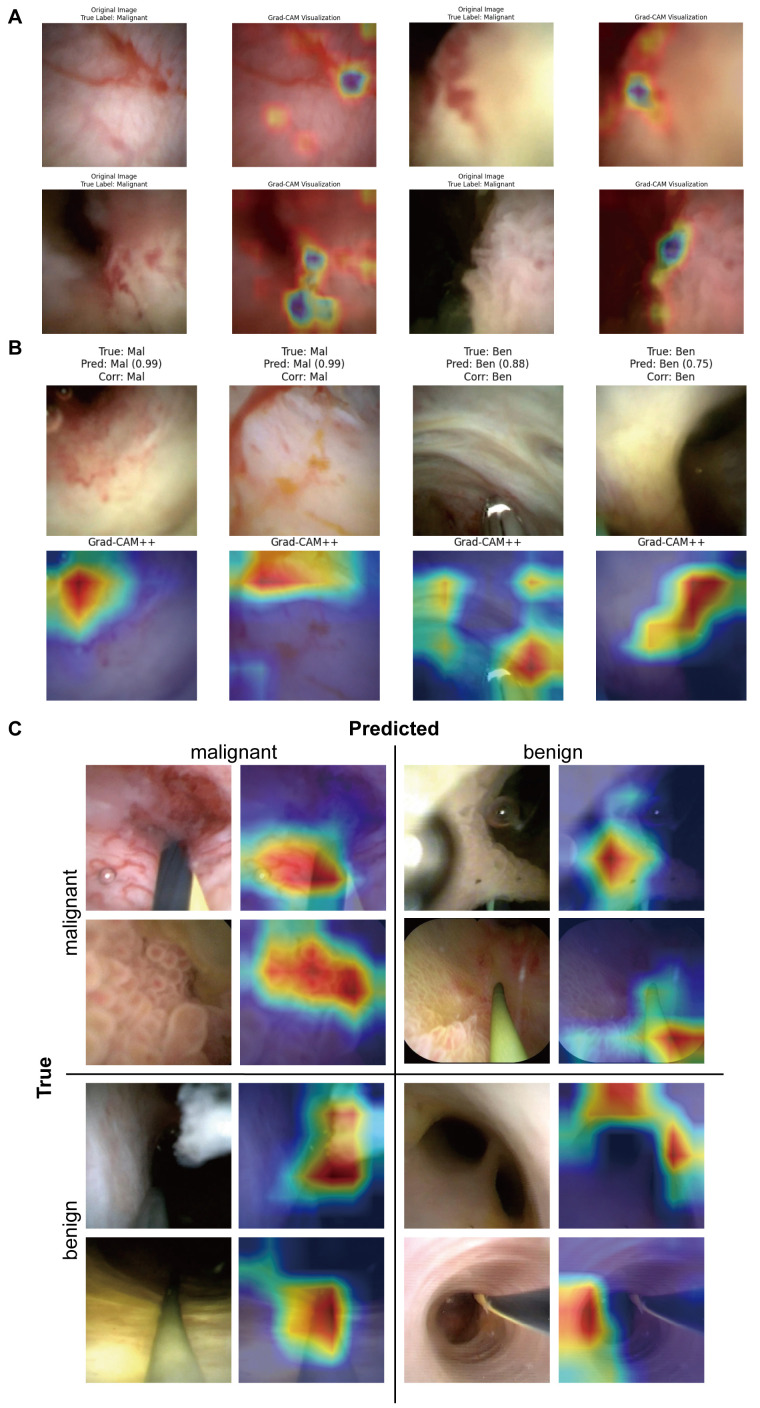
Visualization and representative classification outcomes of the deep learning-based CADe and CADx system. (**A**) Representative attention maps. (**B**) Examples in which attention overlapped with malignant- or benign-appearing morphological regions. (**C**) Grid arranged by reference diagnosis (rows) and predicted diagnosis (columns): upper-left, true positive; upper-right, false negative; lower-left, false positive; lower-right, true negative. Representative errors were associated with limited visualization, instrument occlusion, inflammatory change, or attention to non-lesion artifacts.

Grad-CAM and Grad-CAM++ were used to visualize image regions contributing to CADx predictions (Figure 3A,B). Attention maps frequently overlapped with the three morphological features localized by CADe. Figure 3C is arranged by reference diagnosis (rows) and predicted diagnosis (columns): true-positive, false-negative, false-positive, and true-negative examples occupy the upper-left, upper-right, lower-left, and lower-right quadrants, respectively. In the representative false-negative examples, instrument or sheath occlusion, limited visualization, and tangential views reduced visible malignant mucosal features. Representative false-positive examples showed inflammatory irregularity or attention to non-lesion artifacts. These examples illustrate plausible failure modes and do not constitute a causal classification of all errors.

In the exploratory reader experiment, the CADx model and expert readers showed the same descriptive sensitivity estimate (94.1%). The model’s descriptive specificity estimate was 88.2%, compared with 64.1% for the readers (Table S3). Because the readers also viewed 5 s clips whereas the model used only the static images, these results are not an identical-input head-to-head test and no claim of statistical superiority is made (Figure S3).

### 3.4. Online Demo of the DL-Based MBS CADe and CADx System

The online demonstration processed DSOC clips and displayed annotated outputs with bounding boxes and diagnostic predictions (Video S1). It was not prospectively evaluated as a clinical decision-support system and was not used to collect or reuse third-party data for the present study.

## 4. Discussion

Early and accurate differentiation of MBS from benign counterparts remains clinically challenging. ERCP with brush cytology exhibits low sensitivity, ranging from 20% to 50% [3,29]. While DSOC has improved diagnostic performance with a sensitivity of around 64.3% to 74.0%, substantial interobserver variability persists [30]. Prior DSOC AI studies have included static-image, video-based, and real-time systems for binary neoplasia classification [12,13,14,15,16,17,18,19,20]. Remaining needs include robust external patient-level evaluation and transparent linkage of model predictions to recognized cholangioscopic morphology.

We present a two-stage framework that combines localization of predefined malignancy-associated cholangioscopic features with image-level diagnostic classification. Internal validation showed strong feature-detection and classification performance. In the small external cohort, frame-level AUC was 0.843 and specificity remained high, but frame-level sensitivity was 52.0%. Patient-level aggregation yielded a sensitivity of 85.7%, accuracy of 92.0%, and AUC of 0.881, but these estimates were based on only seven neoplastic and 18 non-neoplastic patients and had wide confidence intervals. They cannot be directly compared with the internal frame-level estimate and do not establish multicenter generalizability. Zhang et al. reported a real-time interpretable binary diagnostic model, and Robles-Medranda et al. developed a multicenter CNN for neoplasia classification [13,15]. The principal distinction of our framework is the addition of a CADe stage that explicitly detects and localizes three named CRM/Mendoza morphological features with bounding boxes before CADx classification and attention mapping. This functional difference is intended to improve interpretability. It is not evidence of superior diagnostic performance.

The marked reduction in external frame-level sensitivity warrants cautious interpretation. Although the external AUC of 0.843 suggests that some discriminatory information was retained, the operating threshold selected on the development cohort may not have been optimally calibrated for external images. Variation in equipment, illumination, color balance, resolution, irrigation, frame selection, and case mix may have contributed, and overfitting cannot be excluded. Standardized center-specific acquisition metadata were not available, so the relative contributions of these factors could not be quantified. Inflammatory strictures remain difficult because reactive neovascularization, mucosal edema, and surface irregularity may resemble neoplasia [31], whereas subtle neoplastic lesions may be flat or obscured. Only four patients had documented IgG4-related sclerosing cholangitis, and no PSC was documented among patients with available history. Reliable discrimination in these subgroups has not been established.

Several limitations should be considered. First, this retrospective study used archived recordings, a modest development cohort, and one patient-level internal validation split without cross-validation. The internal estimate may therefore be unstable or optimistic. Second, use of surgical pathology in 107 patients and a composite clinical reference standard in 42 patients creates potential differential verification and reference-standard misclassification bias, including possible occult malignancy in the follow-up-verified subgroup. Third, frame selection and quality control were manual. The proposed sharpness, exposure, and saturation gates were not calibrated or validated. Fourth, the external cohort included only 25 patients, of whom seven were neoplastic, and standardized center-level acquisition metadata were unavailable. Fifth, few IgG4-related strictures and no documented PSC, pancreatic cancer-related strictures, or metastatic biliary strictures were represented. Finally, the online platform remains a research demonstration. The present ethics approval does not cover retention or secondary training use of third-party uploads. Prospective multicenter evaluation using uncurated procedural videos, broader etiologies, patient-level endpoints, automated quality control, and documented data governance is required before clinical deployment.

## 5. Conclusions

We developed a two-stage CADe/CADx system that combines YOLOv11-based localization of malignancy-associated DSOC features with ResNet18-based diagnostic classification and interpretable attention maps. Internal performance was strong, but external frame-level sensitivity decreased substantially in a small cohort of 25 patients. The present study supports further evaluation of the framework but does not establish multicenter generalizability. Larger prospective studies using uncurated videos, patient-level endpoints, diverse inflammatory and malignant etiologies, and clearly governed data pipelines are required before clinical deployment.

## Figures and Tables

**Table 1:** Baseline characteristics.

Characteristic	Total (*n* = 149)	Neoplasia (*n* = 74)	Non-Neoplasia (*n* = 75)	*p* Value
**Age, median (IQR), years**	63.0 (54.0–69.0)	65.0 (60.2–70.0)	60.0 (50.5–67.5)	0.002
**Sex**				0.367
Female	60 (40.3%)	33 (44.6%)	27 (36.0%)	
Male	89 (59.7%)	41 (55.4%)	48 (64.0%)	
**Age group**				0.008
<30 years	4 (2.7%)	0 (0.0%)	4 (5.3%)	
30–49 years	18 (12.1%)	5 (6.8%)	13 (17.3%)	
50–59 years	32 (21.5%)	13 (17.6%)	19 (25.3%)	
≥60 years	95 (63.8%)	56 (75.7%)	39 (52.0%)	
**Presenting symptoms**				
Jaundice	81 (54.4%)	52 (70.3%)	29 (38.7%)	<0.001
Pruritus	19 (12.8%)	13 (17.6%)	6 (8.0%)	0.091
Abdominal pain	58 (38.9%)	28 (37.8%)	30 (40.0%)	0.867
Weight loss	56 (37.6%)	30 (40.5%)	26 (34.7%)	0.501
**Laboratory parameters, median (IQR) [available *n*]**				
Total bilirubin, umol/L	29.9 (17.9–69.9) [149]	42.5 (20.2–163.5) [74]	27.3 (15.7–41.2) [75]	<0.001
ALP, U/L	201.0 (143.0–365.0) [149]	217.5 (147.8–368.8) [74]	187.0 (137.5–355.0) [75]	0.410
GGT, U/L	206.0 (99.8–432.8) [148]	189.0 (88.0–573.2) [74]	216.0 (109.0–346.2) [74]	0.921
ALT, U/L	69.0 (34.0–125.0) [149]	69.0 (33.0–130.8) [74]	68.0 (36.5–123.0) [75]	0.896
AST, U/L	51.0 (29.0–79.0) [149]	49.0 (33.0–90.5) [74]	52.0 (28.0–71.5) [75]	0.145
AFP, ng/mL	2.7 (2.1–3.8) [116]	2.7 (2.1–3.6) [67]	2.7 (2.0–4.0) [49]	0.978
CEA, ng/mL	2.3 (1.6–4.3) [121]	2.8 (1.7–4.9) [72]	2.0 (1.6–2.8) [49]	0.007
CA19–9, U/mL	59.9 (23.8–218.9) [115]	152.5 (37.3–538.5) [65]	27.8 (15.0–81.5) [50]	<0.001
CA125, U/mL	15.4 (10.6–28.2) [120]	17.6 (12.0–34.7) [70]	13.0 (9.6–19.7) [50]	0.014
**Diagnosis**				
**Neoplastic etiologies**				
Hilar cholangiocarcinoma	39 (26.2%)	39 (52.7%)	0	
Distal cholangiocarcinoma	22 (14.8%)	22 (29.7%)	0	
Gallbladder carcinoma-related stricture	4 (2.7%)	4 (5.4%)	0	
Intrahepatic cholangiocarcinoma	3 (2.0%)	3 (4.1%)	0	
Multiple recurrent biliary malignancies	3 (2.0%)	3 (4.1%)	0	
Cholangiocarcinoma, site unspecified	3 (2.0%)	3 (4.1%)	0	
**Non-neoplastic etiologies**				
Stone-associated stricture	28 (18.8%)	0	28 (37.3%)	
Postoperative stricture	20 (13.4%)	0	20 (26.7%)	
Other inflammatory strictures	17 (11.4%)	0	17 (22.7%)	
IgG4-related sclerosing cholangitis	4 (2.7%)	0	4 (5.3%)	
Benign biliary neoplasm	2 (1.3%)	0	2 (2.7%)	
Congenital biliary dilatation	1 (0.7%)	0	1 (1.3%)	
Indeterminate benign stricture	3 (2.0%)	0	3 (4.0%)	
**Stricture location**				
Cystic duct	5 (3.4%)	2 (2.7%)	3 (4.0%)	1.000
Intrahepatic bile ducts	14 (9.4%)	5 (6.8%)	9 (12.0%)	0.401
Hilar region	63 (42.3%)	44 (59.5%)	19 (25.3%)	<0.001
Common bile duct	57 (38.3%)	29 (39.2%)	28 (37.3%)	0.867
Bilioenteric anastomosis	20 (13.4%)	3 (4.1%)	17 (22.7%)	0.001
Diffuse biliary stricture	8 (5.4%)	2 (2.7%)	6 (8.0%)	0.276
**Reference standard**				<0.001
Surgical histopathological confirmation	107 (71.8%)	66 (89.2%)	41 (54.7%)	
Composite clinical reference standard (≥6-month follow-up)	42 (28.2%)	8 (10.8%)	34 (45.3%)	

IQR, interquartile range; ALP, alkaline phosphatase; GGT, gamma-glutamyl transferase; ALT, alanine aminotransferase; AST, aspartate aminotransferase; AFP, alpha-fetoprotein; CEA, carcinoembryonic antigen; CA19-9, carbohydrate antigen 19-9; CA125, carbohydrate antigen 125. Diagnostic categories are mutually exclusive and reflect the final reference diagnosis rather than image-level malignant-appearing features. No Primary Sclerosing Cholangitis was documented among 146 patients with available history. Location categories are not mutually exclusive. Laboratory values available only as nonnumeric or censored text were treated as unavailable. The numbers of available records are shown in square brackets. Boldface indicates variable-group and category headings.

**Table 2:** Performance metrics of the DL-based MBS CADe and CADx system in diagnosis classification for MBS during DSOC.

Metrics	Internal Validation	External Validation
AUC (95% CI)	0.960 (0.945–0.975)	0.843 (0.826–0.864)
Sensitivity (95% CI)	0.930 (0.898–0.953)	0.520 (0.344–0.620)
Specificity (95% CI)	0.820 (0.758–0.869)	0.952 (0.918–0.969)
Accuracy (95% CI)	0.892 (0.862–0.916)	0.887 (0.845–0.916)
PPV (95% CI)	0.907 (0.872–0.933)	0.659 (0.488–0.777)
NPV (95% CI)	0.862 (0.803–0.906)	0.918 (0.890–0.935)

AUC, area under the curve; CI, confidence interval; PPV, positive predictive value; NPV, negative predictive value. Metrics are frame-level. Internal proportion confidence intervals are Wilson intervals; external confidence intervals were obtained by stratified patient-cluster bootstrap with 1000 resamples.

## Data Availability

The source code for the CADe/CADx system and online demonstration is publicly available at https://github.com/ZizhanT/dsoc-lesion-detection (accessed on 23 July 2026). The patient imaging datasets are not publicly available because of patient privacy and institutional data-protection requirements but may be available from the corresponding author upon reasonable request and subject to institutional approval. No third-party uploaded videos were used for model development, evaluation, or secondary training in this study. Any future collection or reuse of external uploads would require separate ethics approval and an appropriate consent or waiver framework.

## Supplementary Materials

The following supporting information can be downloaded at https://www.mdpi.com/article/10.3390/cancers18152410/s1. Figure S1: (A,B) Training process and (C) representative examples of the computer-aided detection model; Figure S2: Patient-level external validation; Figure S3: (A) Visualizations of feature maps. (B) Exploratory descriptive comparison with expert endoscopists; Table S1: Performance metrics of the DL-based MBS CADe and CADx system in detection of malignancy-associated morphological features during DSOC; Table S2: Baseline characteristics of external validation cohort; Table S3: Exploratory descriptive diagnostic-performance comparison between expert endoscopists and the CADx model; Table S4: Frame- and patient-level performance metrics of the DL-based MBS CADx system in the external validation cohort; Table S5: Comparative performance of candidate object-detection architectures for the CADe stage; Video S1: Demonstration of the CADe/CADx system.

## Abbreviations

AI	Artificial Intelligence
AUC	Area Under the Curve
CADe	Computer-Aided Detection
CADx	Computer-Aided Diagnosis
CI	Confidence Interval
CNN	Convolutional Neural Network
CRM	Carlos Robles-Medranda (Classification)
DL	Deep Learning
DSOC	Digital Single-Operator Cholangioscopy
ERCP	Endoscopic Retrograde Cholangiopancreatography
Grad-CAM	Gradient-Weighted Class Activation Mapping
IPMN-B	Intraductal Papillary Mucinous Neoplasm of the Bile Duct
IQR	Interquartile Range
mAP50	Mean Average Precision at IoU 0.50
MBS	Malignant Biliary Stricture
NPV	Negative Predictive Value
PPV	Positive Predictive Value
PSC	Primary Sclerosing Cholangitis
ResNet18	Residual Network-18
YOLOv11	You Only Look Once version 11
