# Supplementary material for: Deep Learning-Based Computer-Aided Detection and Diagnosis System for Malignant Biliary Stricture (With Video)
Source: Cancers (Basel). 2026 Jul 26;18(15):2410. doi: 10.3390/cancers18152410 (PMC13465266; doi:10.3390/cancers18152410)
Supplement: Supplementary file 1 [file cancers-18-02410-s001.zip › cancers-4428315-supplementary.pdf]

Supplementary Material

Deep Learning-Based Computer-Aided Detection and Diagnosis System for Malignant Biliary Stricture (With Video)

Qingyu Tang [1], Sanping Zhou [2], Zizhan Tang [3], Kangpeng Li [1], Zejian Huang [4], Lei Zhang [4], Dapeng Bian [5], Qiushi Feng [5], Qi Li [1], Hao Sun [1], Jie Tao [1], Le Wang [2], Zhimin Geng [1*] and Chen Chen [1*]

Table S1. Performance metrics of the DL-based MBS CADe and CADx system in detection of malignancy-associated morphological features during DSOC.

Malignancy classes	Precision	Recall	mAP50	mAP50-95
All	0.920 (0.900 – 0.937)	0.870 (0.844 – 0.893)	0.912	0.787
Nodular protrusion	0.936 (0.915 – 0.953)	0.947 (0.928 – 0.962)	0.953	0.866
Surface irregularity	0.926 (0.904 – 0.944)	0.878 (0.851 – 0.902)	0.933	0.804
Vascular irregularity	0.898 (0.873 – 0.919)	0.784 (0.749 – 0.817)	0.851	0.689

mAP50, mean average precision at 50% intersection-over-union; IoU, intersection-over-union.

Table S2. Baseline characteristics of external validation cohort.

	Total (n=25)	Neoplasia (n=7)	Non-Neoplasia (n=18)	P value
Sex				0.075
Female	15 (60.0%)	2 (28.6%)	13 (72.2%)	
Male	10 (40.0%)	5 (71.4%)	5 (27.8%)	
Age, median (IQR), years				
Female	59.3 (55.0 – 71.0)	73.5 (68.0 – 79.0)	57.2 (55.0 – 71.0)	
Male	61.6 (57.0 – 75.0)	67.2 (58.5 – 76.0)	56 (41.5 – 69.5)	
Age group				0.488
< 30 y	1 (4.0%)	0 (0.0%)	1 (5.6%)	1.000
30 – 49 y	3 (12.0%)	0 (0.0%)	3 (16.7%)	0.534
50 – 59 y	5 (20.0%)	1 (14.3%)	4 (22.2%)	1.000
≥ 60 y	16 (64.0%)	6 (85.7%)	10 (55.6%)	0.355
Diagnosis				<0.001
Stone	8 (32.0%)	0 (0.0%)	8 (44.4%)	0.057
Inflammation	4 (16.0%)	0 (0.0%)	4 (22.2%)	0.295
Postoperative biliary stricture	6 (24.0%)	0 (0.0%)	6 (33.3%)	0.137
GBC	1 (4.0%)	1 (14.3%)	0 (0.0%)	0.280
HCCA	6 (24.0%)	6 (85.7%)	0 (0.0%)	<0.001
Location of stenosis				0.004
Cystic duct	1 (4.0%)	1 (14.3%)	0 (0.0%)	0.280
Intrahepatic	2 (8.0%)	0 (0.0%)	2 (11.1%)	1.000
Hilum	8 (32.0%)	6 (85.7%)	2 (11.1%)	0.001
Common bile duct	1 (4.0%)	0 (0.0%)	1 (5.6%)	1.000
Anastomosis	6 (24.0%)	0 (0.0%)	6 (33.3%)	0.137
Diffuse biliary stricture	7 (28.0%)	0 (0.0%)	7 (38.9%)	0.133

IQR, interquartile range; HCCA, hilar cholangiocarcinoma; DCC, distal cholangiocarcinoma; GBC, gallbladder carcinoma.

Table S3. Exploratory descriptive diagnostic-performance comparison between expert endoscopists and the CADx model.

	Sensitivity, % (95% CI)	Specificity, % (95% CI)	PPV, % (95% CI)	NPV, % (95% CI)
Endoscopists	94.1 (89.5 – 96.8)	64.1 (53.8 – 73.4)	82.8 (77.0 – 87.5)	85.5 (75.3 – 92.2)
CADe and CADx system	94.1 (89.6 – 96.9)	88.2 (80.2 – 93.4)	93.6 (88.9 – 96.5)	89.1 (81.3 – 94.2)

CI, confidence interval; PPV, positive predictive value; NPV, negative predictive value.

Table S4. Frame- and patient-level performance metrics of the DL-based MBS CADx system in the external validation cohort.

Metrics	External validation (frame-level)	External validation (patient-level)
AUC (95% CI)	0.843 (0.826–0.864)	0.881 (0.619–1.000)
Sensitivity (95% CI)	0.520 (0.344–0.620)	0.857 (0.487–0.974)
Specificity (95% CI)	0.952 (0.918–0.969)	0.944 (0.742–0.990)
Accuracy (95% CI)	0.887 (0.845–0.916)	0.920 (0.750–0.978)
PPV (95% CI)	0.659 (0.488–0.777)	0.857 (0.487–0.974)
NPV (95% CI)	0.918 (0.890–0.935)	0.944 (0.742–0.990)

AUC, area under the curve; CI, confidence interval; PPV, positive predictive value; NPV, negative predictive value.

Table S5. Comparative performance of candidate object-detection architectures for the CADe stage.

Model	Feature class	Precision	Recall	mAP50	mAP50–95
YOLOv5	All	0.765	0.778	0.820	0.521
	Nodular protrusion	0.770	0.857	0.874	0.625
	Surface irregularity	0.793	0.768	0.823	0.481
	Vascular irregularity	0.733	0.709	0.762	0.456
YOLOv8	All	0.619	0.708	0.695	0.426
	Nodular protrusion	0.836	0.941	0.927	0.691
	Surface irregularity	0.609	0.660	0.650	0.349
	Vascular irregularity	0.412	0.524	0.507	0.238
**YOLOv11**	**All**	**0.920**	**0.870**	**0.912**	**0.787**
	Nodular protrusion	0.936	0.947	0.953	0.866
	Surface irregularity	0.926	0.878	0.933	0.804
	Vascular irregularity	0.898	0.784	0.851	0.689
YOLOv12	All	0.861	0.595	0.755	0.457
	Nodular protrusion	0.865	0.752	0.866	0.680
	Surface irregularity	0.897	0.548	0.732	0.378
	Vascular irregularity	0.821	0.484	0.667	0.312

CADe, computer-aided detection; mAP50, mean average precision at an intersection-over-union threshold of 0.50; mAP50–95, mean average precision averaged across intersection-over-union thresholds from 0.50 to 0.95.

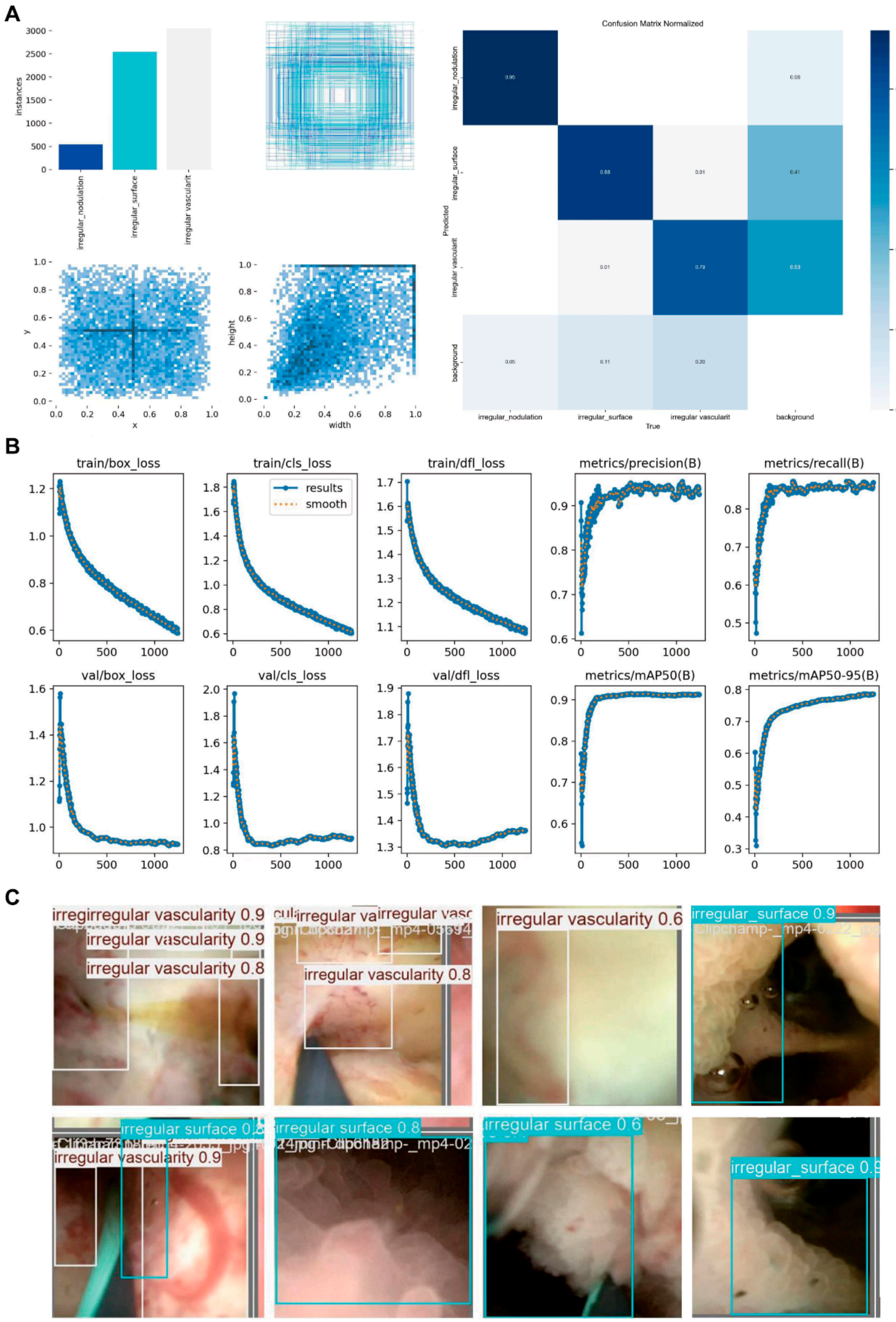

Figure S1. (A, B) Training process and (C) representative examples of the computer-aided detection model.

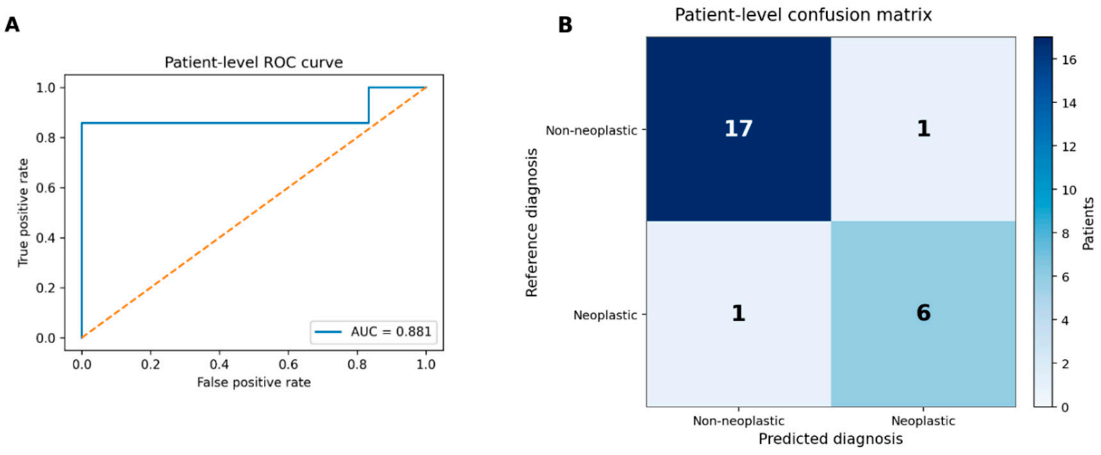

Figure S2. Patient-level external validation. (A) Receiver operating characteristic curve (AUC = 0.881, 95% CI 0.619–1.000). (B) Confusion matrix (17 true negatives, 1 false positive, 1 false negative, and 6 true positives). Patient-level predictions were derived as the mean of the top-scoring decile of frame probabilities per patient and classified using the fixed internal-validation threshold of 0.58.

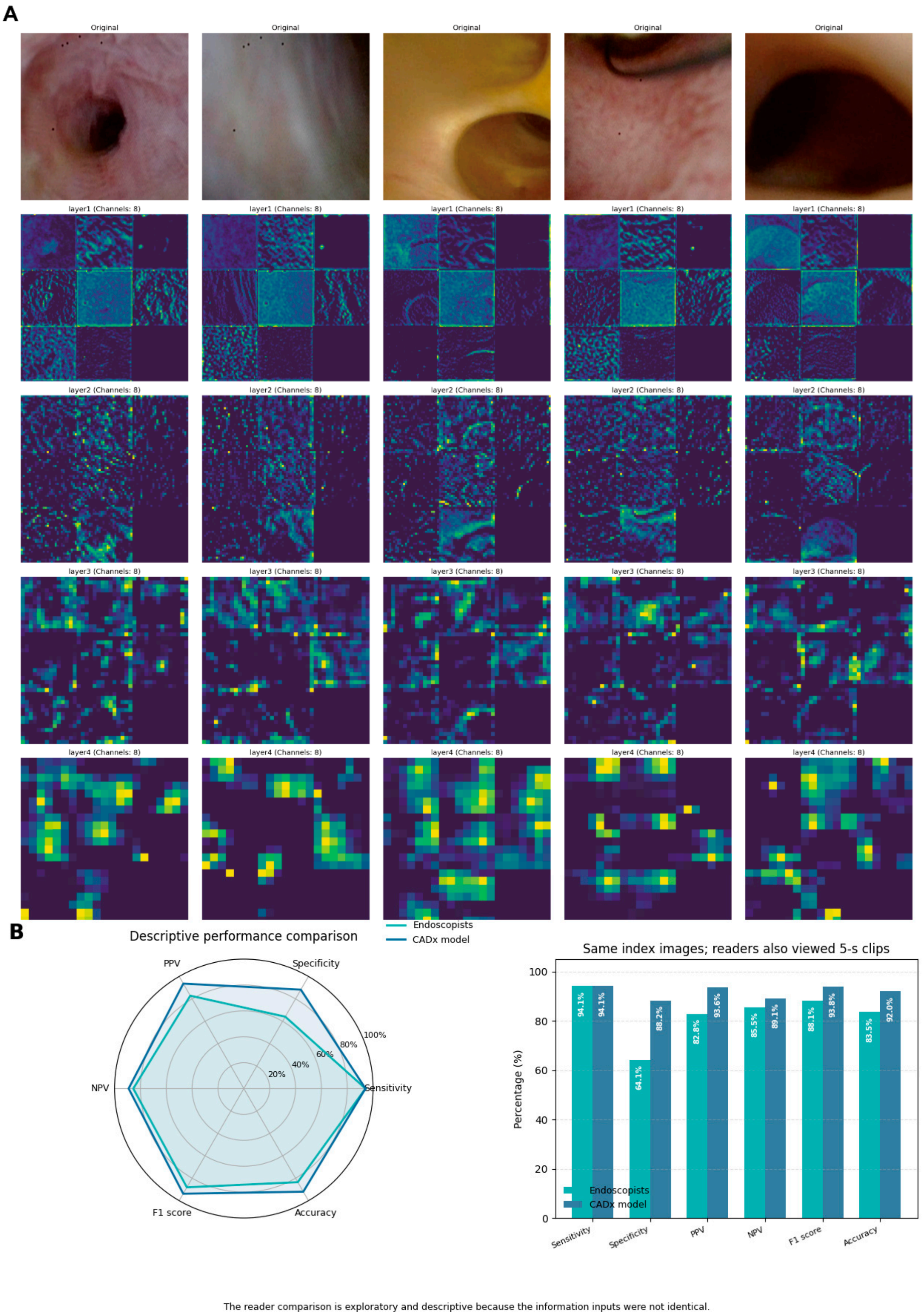

Figure S3. (A) Visualizations of feature maps. (B) Exploratory descriptive comparison with expert endoscopists. The same index images were assessed, but readers additionally viewed corresponding 5-second clips; no formal superiority inference is made.
